# A Multi-Center Randomised Controlled Trial of Gatifloxacin versus Azithromycin for the Treatment of Uncomplicated Typhoid Fever in Children and Adults in Vietnam

**DOI:** 10.1371/journal.pone.0002188

**Published:** 2008-05-21

**Authors:** Christiane Dolecek, Tran Thi Phi La, Nguyen Ngoc Rang, Le Thi Phuong, Ha Vinh, Phung Quoc Tuan, Doan Cong Du, Nguyen Thi Be Bay, Duong Thanh Long, Luong Bich Ha, Nguyen Trung Binh, Nguyen Thi Anh Hong, Pham Ngoc Dung, Mai Ngoc Lanh, Phan Van Be Bay, Vo Anh Ho, Nguyen Van Minh Hoang, Tran Thu Thi Nga, Tran Thuy Chau, Constance Schultsz, Sarah J. Dunstan, Kasia Stepniewska, James Ian Campbell, To Song Diep, Buddha Basnyat, Nguyen Van Vinh Chau, Nguyen Van Sach, Nguyen Tran Chinh, Tran Tinh Hien, Jeremy Farrar

**Affiliations:** 1 Oxford University Clinical Research Unit, The Hospital for Tropical Diseases, Ho Chi Minh City, Vietnam; 2 The Hospital for Tropical Diseases, Ho Chi Minh City, Vietnam; 3 An Giang Provincial Hospital, Long Xuyen, Vietnam; 4 Dong Thap Provincial Hospital, Cao Lanh, Dong Thap, Vietnam; 5 Nuffield Department of Clinical Medicine, John Radcliffe Hospital, Oxford, United Kingdom; 6 The London School of Hygiene and Tropical Medicine, London School of Hygiene and Tropical Medicine, London, United Kingdom; 7 Patan Hospital, Lagankhel, Lalitpur, Nepal; Cincinnati Children's Hospital Medical Center, United States of America

## Abstract

**Background:**

Drug resistant typhoid fever is a major clinical problem globally. Many of the first line antibiotics, including the older generation fluoroquinolones, ciprofloxacin and ofloxacin, are failing.

**Objectives:**

We performed a randomised controlled trial to compare the efficacy and safety of gatifloxacin (10 mg/kg/day) versus azithromycin (20 mg/kg/day) as a once daily oral dose for 7 days for the treatment of uncomplicated typhoid fever in children and adults in Vietnam.

**Methods:**

An open-label multi-centre randomised trial with pre-specified per protocol analysis and intention to treat analysis was conducted. The primary outcome was fever clearance time, the secondary outcome was overall treatment failure (clinical or microbiological failure, development of typhoid fever-related complications, relapse or faecal carriage of *S. typhi).*

**Principal Findings:**

We enrolled 358 children and adults with suspected typhoid fever. There was no death in the study. 287 patients had blood culture confirmed typhoid fever, 145 patients received gatifloxacin and 142 patients received azithromycin. The median FCT was 106 hours in both treatment arms (95% Confidence Interval [CI]; 94–118 hours for gatifloxacin *versus* 88–112 hours for azithromycin), (logrank test *p* = 0.984, HR [95% CI] = 1.0 [0.80–1.26]).

Overall treatment failure occurred in 13/145 (9%) patients in the gatifloxacin group and 13/140 (9.3%) patients in the azithromycin group, (logrank test *p* = 0.854, HR [95% CI] = 0.93 [0.43–2.0]). 96% (254/263) of the *Salmonella enterica* serovar Typhi isolates were resistant to nalidixic acid and 58% (153/263) were multidrug resistant.

**Conclusions:**

Both antibiotics showed an excellent efficacy and safety profile. Both gatifloxacin and azithromycin can be recommended for the treatment of typhoid fever particularly in regions with high rates of multidrug and nalidixic acid resistance. The cost of a 7-day treatment course of gatifloxacin is approximately one third of the cost of azithromycin in Vietnam.

**Trial Registration:**

Controlled-Trials.com ISRCTN67946944

## Introduction

There are approximately 21 million cases of typhoid fever annually, with more than 210 000 deaths [1]. The emergence of antimicrobial drug resistance in *Salmonella enterica* serovar Typhi (*S. typhi*) is a major problem particularly in South East Asia and the Indian sub-continent and challenges our current treatment options [2]–[4]. There is a need for an efficacious, safe and affordable oral treatment, particularly in regions with a high proportion of both multidrug and nalidixic acid resistant *S. typhi*.

In Vietnam, multidrug resistant (MDR) isolates of *S. typhi* (resistant to ampicillin, chloramphenicol and trimethoprim-sulfamethoxazol) first appeared in 1993 [5]. From this time the fluoroquinolones became the treatment of choice for typhoid fever [4], and were simultaneously sold widely over the counter to treat fever of various aetiologies. The extensive antibiotic pressure lead to the selection of single point mutations in the DNA *Gyrase A* of *S. typhi*, causing resistance to nalidixic acid (the prototype quinolone) and reduced susceptibility to the fluoroquinolones (but formally these isolates are still within the Clinical Laboratory Standard Institute (CLSI) breakpoints for susceptibility) [6]. This resulted in a poor clinical response to treatment with the older generation fluoroquinolones, ofloxacin or ciprofloxacin [7], [8].

The World Health Organisation recommends the fluoroquinolones or cefixime for the treatment of MDR typhoid fever and azithromycin, the third-generation cephalosporins, or a 10–14 day course of high-dose older generation fluoroquinolones (e.g. ofloxacin or ciprofloxacin) for the treatment of nalidixic acid resistant typhoid fever [9].

Azithromycin, an azalid antibiotics, has achieved excellent clinical results in the treatment of MDR and nalidixic acid resistant typhoid fever [7], [8]. However azithromycin is expensive. Cefixime has recently failed in the treatment of nalidixic acid resistant typhoid fever in Nepal (these data were not available at the start of this trial) [10].

A recent trial from southern Vietnam used ofloxacin at the maximum recommended dose of 20 mg/kg/day for 7 days for the treatment of MDR and nalidixic acid resistant typhoid fever and showed high clinical failure rates (36%), high immediate post-treatment faecal carriage (19%), which may lead to transmission in the community after discharge from hospital, and prolonged mean fever clearance times of 8.2 days (95% CI, 7.2–9.2 days) [8].

These results underline that the older generation fluoroquinolones are clearly failing in the treatment of nalidixic acid resistant typhoid fever.

Of the newer fluoroquinolones, gatifloxacin is available and affordable in South and South East Asia including Vietnam [10]. Of all the fluoroquinolones, gatifloxacin showed the lowest minimum inhibitory concentrations (MICs) for nalidixic acid resistant *S. typhi* from Nepal [11] and Vietnam and a rapid bactericidal effect in time-kill experiments involving *S. typhi* isolates with single and double mutations in the *GyrA* of *S. typhi* [6].

We conducted a randomised controlled trial comparing the efficacy of gatifloxacin to azithromycin in southern Vietnam, an area characterised by a very high proportion of MDR (88%) and nalidixic acid resistant (93%) *S. typhi* isolates [8].

## Methods

The protocol for this trial and supporting CONSORT checklist are available as supporting information; see Checklist S1 and Protocol S1.

### Study design and objectives

The study was designed as a multicenter, open-label randomised controlled trial to compare the efficacy and safety of gatifloxacin versus azithromycin for the treatment of uncomplicated typhoid fever in children and adult in-patients in southern Vietnam.

The overall objective of the study was to identify an efficacious, safe, available and affordable oral treatment for MDR and nalidixic acid resistant typhoid fever.

### Participants

Patients were eligible to be included in the study if they had clinically suspected or culture confirmed uncomplicated typhoid fever and if fully informed written consent had been obtained. For children, consent was obtained from the parent. Exclusion criteria were pregnancy, age under 6 months, history of hypersensitivity to either of the trial drugs, any signs of severe typhoid fever (shock, deep jaundice, encephalopathy, convulsions, bleeding, suspicion or evidence of gut perforation), or previous reported treatment with a fluoroquinolone antibiotics, a third generation cephalosporine or macrolide antibiotics within one week prior to hospital admission.

### The study sites and ethical approval

The study was conducted at three hospitals in the south of Vietnam.

Adult and paediatric patients were recruited at the Hospital for Tropical Diseases in Ho Chi Minh City, at the Dong Thap Provincial hospital in Cao Lanh, Dong Thap province and at the An Giang Provincial hospital in Long Xuyen, An Giang province.

The study was approved by the Ethical and Scientific Committee of the Hospital for Tropical Diseases in Ho Chi Minh City and the Oxford University Tropical Research Ethics Committee (OXTREC), UK for all three study sites. The clinical and microbiological data from the first 40 patients recruited to each arm of the study were sent to the independent Data Safety and Monitoring Committee for their advice regarding the continuation of the study. The study was not stopped.

### Intervention

According to their randomisation number patients were assigned to oral treatment with either 20 mg/kg azithromycin (Zithromax® suspension, Pfizer, USA; 200 mg/5 mL or Zithromax® tablets, Pfizer, USA; 500 mg/tablet) or 10 mg/kg gatifloxacin (Tequin®, Bristol-Myers Squibb, USA; 400 mg/tablet) once daily for 7 days. Tablets were cut to obtain the appropriate study dosage and administered with water. Inevitably, the dose administered was an estimate of 10 mg/kg/day of gatifloxacin or 20 mg/kg/day of azithromycin (number of tablets or proportions of tablets were documented in the CRFs). Gatifloxacin was only available as tablets, which were cut to obtain the appropriate dosage and crushed if necessary for children.

The maximum dose of azithromycin was 1 g per day. All drugs were purchased commercially.

### Procedures

#### In-patient procedures

On admission to the hospital the patient's full history was taken, a standard clinical examination was performed and axillary temperature, weight and height were measured. Before treatment, full blood counts including white blood differential counts, serum aspartate transaminase (AST), serum alanine transaminase (ALT) and bilirubin were checked and blood cultures were obtained. For adult patients, creatinine, blood urea nitrogen (BUN) and glucose levels were additionally measured. In some patients bone marrow cultures were obtained. Urines were checked with dipstick and pre-treatment stool cultures were obtained. Chest X-ray and abdominal ultrasound were performed and repeated as clinically indicated. Randomisation and initiation of therapy took place either immediately on admission to hospital or patients were observed until results of blood tests including blood cultures were available and then randomised. Vital signs including measurement of axillary temperatures were measured and recorded every 6 hours (at 6, 12, 18 and 24 hours) until discharge. Patients were examined daily until discharge from hospital, with particular reference to clinical symptoms, FCT, side effects of the drug and any complication of the disease. Additionally laboratory tests were scheduled if clinically indicated. All adverse events were recorded. On day 7 to 9 after the start of treatment full blood counts, liver function tests, blood and stool cultures were checked. In case of insufficient response to therapy, development of complications or drug-associated adverse events, the initial treatment was suspended and parenteral ceftriaxone (2 g per day) in two divided doses was used as rescue treatment for 10 days.

#### Follow-up procedures

Out-patient follow-up appointments were scheduled at 1 month, 3 months and 6 months after discharge from hospital to seek evidence for relapse (1 month visit) and check for chronic typhoid carriage (all visits). At these appointments a full history was taken, relevant examinations performed and stool cultures obtained. Blood or bone marrow cultures were only obtained if clinical symptoms were indicative of acute infection. If patients did not attend their follow up appointment, they were reminded by letter or a member of the study team visited their home. If stool samples were not available, a rectal swab was obtained.

Patients with convalescent stool carriage of *S. typhi* or *S. paratyphi* A were retreated according to the sensitivity of the isolate and were further followed up. Ultrasound was performed to exclude biliary or kidney stones if carriage was persistent.

### Microbiology

Five to 8 mL of blood was collected from adults and inoculated into Bactec Plus Aerobic Blood bottles, and 3 to 5 mL of blood from children was inoculated into Bactec Peds Plus culture bottles (Becton Dickinson, New Jersey, USA). The bottles were incubated at 37°C in the BACTEC 9050 automated analyser for 7 days and sub-cultured according to standard methods when the machine indicated a positive signal, or incubated at 37°C in a standard laboratory incubator (An Giang hospital) and examined daily.

Stool samples or rectal swabs were inoculated onto MacConkey agar and Xylose Lysine Decarboxylase (XLD) agar plates, and in 10 mL of selenite F broth. Plates and broth were incubated at 37°C overnight and the broth was sub-cultured on MacConkey and XLD agar plates the next morning.

Isolates were screened using standard biochemical tests and *S. typhi* and *S. paratyphi* A were identified using API20E (bioMerieux, Paris, France) and slide agglutination with specific antisera (Murex, Dartford, UK).

Antimicrobial susceptibility testing was performed by disc diffusion according to Clinical Laboratory Standards Institute (CLSI) guidelines [12], using CLSI breakpoints [13]. Antimicrobial agents tested were: ampicillin, chloramphenicol, trimethoprim-sulfamethoxazol, nalidixic acid, ofloxacin, ciprofloxacin and ceftriaxone (Oxoid, Basingstoke, UK). Minimum Inhibitory Concentrations (MICs) for amoxicillin, chloramphenicol, nalidixic acid, ofloxacin, ciprofloxacin, gatifloxacin, ceftriaxon and azithromycin were determined by E-test (AB Biodisk, Solna, Sweden). Multidrug resistance (MDR) of isolates was defined as resistance to chloramphenicol (MIC≥32 µg/mL), ampicillin (MIC≥32 µg/mL) and trimethoprim-sulfamethoxazole (MIC≥8/152 µg/mL). Nalidixic acid resistance was defined as an MIC≥32 µg/mL. The CLSI breakpoints for ofloxacin and gatifloxacin were ≤2 µg/mL susceptible and ≥8 µg/mL resistant, for ciprofloxacin ≤1 µg/mL susceptible and ≥4 µg/mL resistant and for ceftriaxone ≤8 µg/mL susceptible and ≥64 µg/mL resistant. There were no CLSI MIC breakpoints for azithromycin [13]. The control strains used for all susceptibility tests were *E. coli* ATCC 25922, *Pseudomonas aeruginosa* ATCC 27853, *Staphylococcus aureus* ATCC 29213.

All cultures, identification of S. *typhi* and *S. paratyphi* A and disc diffusion were performed at the three study sites. All isolates were sent to the Hospital for Tropical Diseases, Ho Chi Minh City, for confirmation of identity, susceptibility testing and MIC testing.

### Outcomes of the study

The primary endpoint of the study was the resolution of fever (fever clearance time, FCT), which was defined as the time from the start of the antibiotic treatment to when the axillary temperature first fell ≤37.5°C and remained there for at least 48 hours. Secondary endpoints were the overall failure to treatment, which was defined a priori as any of the following: clinical failure (persistence of fever and symptoms two days after the end of treatment, i.e. on day 10) or need for re-treatment due to insufficient treatment response as judged by the treating physician; microbiological failure (positive blood culture on day 7 to 9 after the start of treatment); the development of typhoid fever-related complications during hospital-stay; the occurrence of relapse (symptoms and signs suggestive of typhoid fever) within 1 month after completion of treatment or the detection of faecal carriage of *S. typhi* at the follow-up visits at 1, 3 and 6 months (to exclude faecal carriage a minimum of two consecutive follow-up visits had to be attended).

### Sample Size

The primary outcome measure for the study was the fever clearance time (FCT).

Previous studies that used azithromycin to treat typhoid fever patients, reported a mean fever clearance time of 130 hours [7] and 139 hours [8]. For gatifloxacin, clinical observations from a small number of typhoid fever patients were available and indicated a mean FCT of 76 hours. We calculated that 139 patients with culture-confirmed typhoid fever would be needed in each treatment arm to detect a Hazard Ratio of 1.40 with two-sided alpha of 0.05 and power of 0.80 [14]. Therefore, assuming a median fever clearance time of 130 hours for azithromycin, the sample size of 140 patients with culture-confirmed typhoid fever in each arm would give power of at least 0.80 to detect a difference between treatments if the fever clearance time in the gatifloxacin group was 92 hours or less.

### Randomization procedures and assignment of intervention (sequence generation, allocation concealment, implementation)

An administrator independent from the study generated the random number sequence in Excel using RAND function. These randomised codes were blocked in a size of 50. Treatment assignments were folded and kept in opaque, sealed, sequentially numbered envelopes at all three study sites. Due to logistic reasons randomisation was not stratified by centre.

After all inclusion and exclusion criteria were checked, and informed consent given, the study doctor opened the envelope to determine which treatment the subject would receive. The sealed envelopes were opened in strict numeric sequence.

### Blinding

This study was conducted as an open study.

### Statistical methods

Binary outcomes (clinical failure, microbiological failure, typhoid fever-related complications) were compared between the two treatment groups using Fisher's exact test, assuming the worst case scenario (all lost to follow up treated as failures). The un-adjusted Odds Ratio (OR) and Cornfield's 95% confidence interval [15] were calculated to show the relative risk of developing individual secondary outcomes (clinical, microbiological failure, typhoid fever-related complications) in the gatifloxacin group compared to the azithromycin group.

Fever clearance time, time to relapse and time to overall failure were analysed using survival methods. The time to overall failure equaled the earliest time individual failure was recorded. Kaplan-Meier estimates of probabilities of each event were calculated at any time-point, and they were compared between the two treatment groups using the log-rank test. Data of patients who were lost to follow-up were censored at the time of the last recorded outcome. The Hazard Ratio was derived from Cox proportional hazard model [16].

All patients with positive blood or bone marrow culture for *S. typhi* and *S. paratyphi* A (per protocol analysis) and separately all randomised patients (intention to treat analysis) were analysed.

All data were recorded prospectively into individual Case Record Forms (CRF) and entered into an electronic database (Epi Info 2003, CDC, Atlanta, USA) and double-checked.

Analysis was performed using STATA version 8.0 (Stata Corporation, Texas, USA) statistical software program.

## Results

### Participant flow and recruitment

**Figure 1:**
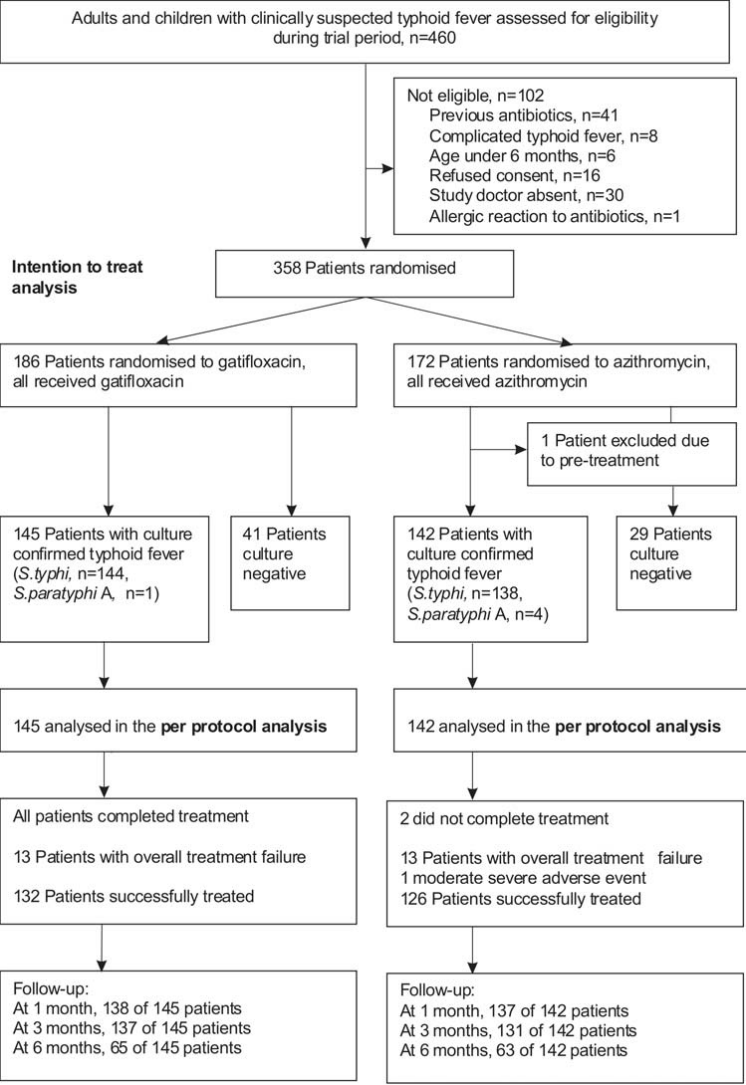
Profile of the Trial.

During the study period, 460 patients were assessed for eligibility (Figure 1). One hundred and two patients were non-eligible, the main reason was the reported previous use of fluoroquinolone, macrolid or third generation cephalosporin antibiotics (41 patients) in the week before hospitalisation.

Between April 2004 and August 2005, 358 patients with suspected typhoid fever were randomised to receive either gatifloxacin or azithromycin. Two hundred eighty-eight of these patients had blood or bone marrow confirmed typhoid fever and 70 patients were culture negative for *S. typhi*. One culture positive patients was excluded from the per protocol analysis (PP), because he had received ciprofloxacin before entry to the trial. The PP group consisted of 287 patients, 145 in the gatifloxacin group and 142 in the azithromycin group. All PP patients, except two in the azithromycin group, finished the full course of treatment.

The total number patients visiting the follow-up at 1 month was 275 out of 287 (96%), at 3 months 268 out of 287 (93%), at 6 months 128 out of 287 (44%) patients.

### Numbers analysed

All 358 randomised patients were analysed in the intention to treat (ITT) analysis. Two hundred and eighty-seven patients with culture confirmed typhoid fever, 145 treated with gatifloxacin and 142 with azithromycin, were analysed in the pre-specified PP analysis.

### Baseline Data

The median age of patients recruited in this trial was 11 years (range 1–41) in the PP group.

The baseline characteristics of the patients were similar in the two treatment groups and in the culture negative patients (Table 1).

**Table 1:** Baseline characteristics of culture confirmed patients (PP analysis) and culture negative patients.

Characteristics	Culture confirmed patients treated with		Blood culture negative patients, n = 70
	Gatifloxacin, n = 145	Azithromycin, n = 142	
Median age in years (range)	11 (2–30)	11 (1–41)	9 (2–42)
Number of children defined as age under 15 (%)	109 (75.2)	101 (71.1)	56 (80)
Number of males (%)	71 (49)	76 (53.5)	29 (41)
Median weight in kilograms (range)	25 (8.5–55)	24.5 (9.5–57)	19.5 (10.5–53)
Median duration of fever before admission in days (range)	7 (2–30)	7 (2–30)	7 (3–30)
Number of patients who received pretreatment (%)*	21 (14.5)	18 (12.7)	16 (22.9)
Median temperature at admission in °C (range)	39 (37–40.5)	39 (37.3–41)	38.75 (37–40)
Hepatomegaly, number of patients (%)	69 (47.6)	63 (44.4)	36 (51.4)
Splenomegaly, number of patients (%)	17 (11.7)	14 (9.8)	2 (2.9)
Abdominal pain, number of patients (%)	82 (56.5)	76 (53.5)	43 (61.4)
Weight loss, number of patients (%)	69 (47.6)	71 (50)	21 (30)
Vomiting, number of patients (%)	47 (32.4)	54 (38)	19 (27.1)
Diarrhoea, number of patients (%)	95 (65.5)	82 (57.7)	49 (70)
Mild jaundice, number of patients (%)	12 (8.3)	20 (14.1)	1 (1.4)
Median haematocrit in % (range)	34.3 (19.2–54.3)	34.6 (20.7–60.5)	34.2 (24.6–46.7)
Median white cell count, 10^9^/L (range)	6.9 (2–17.2)	7.05 (2.4–16.8)	7.25 (2.8–11.7)
Median platelet count, 10^9^/L (95% CI, range)	172 (34–500)	172.5 (45–578)	208 (51–496)
Median AST, U/L(range)	85 (16.9–773)	72 (17.6–1190)	50.1 (11–533)
Median ALT, U/L (range)	67.4 (10.3–276)	59.4 (10.2–734)	44.1 (10–375)
Numbers of *S.typhi*/*S.paratyphi* A isolated from blood cultures	144/1	138/4	0
Positive pretreatment faecal cultures, numbers (%)	11/124 (8.9)	6/118 (5.1)	0

AST, Serum Aspartate Aminotransferase AST (normal range, 12–30 U/L).

ALT, Serum Alanine Aminotransferase ALT (normal range, 13–40 U/L).

*Treatment with amoxicilline or cotrimoxazole prior to hospital admission.

Patients with suspected and blood culture confirmed typhoid fever were eligible for this trial. In the PP group, the median delay in time between hospital admission and randomisation was 3 days (interquartile range 1–4) in the gatifloxacin group and 3 days (interquartile range 2–4) in the azithromycin group. In the ITT group, the median delay in time between hospital admission and randomisation was 2 days (interquartile range 0–4) in the gatifloxacin group and 3 days (interquartile range 1–4) in the azithromycin group.

### Protocol deviations and modifications

At one study site, the An Giang Provincial Hospital, the follow-up visit at 6 months was not possible for logistic reasons. It was therefore agreed to carry out two follow-up visits at 1 and 3 months and to schedule additional (cross-sectional) follow-up dates to invite as many patients as possible to a third follow-up visit. From the PP population, 22 out of 91 patients in the gatifloxacin arm and 17 out of 87 patients in the azithromycin arm attended the third visit.

### Outcomes and estimation

#### Primary outcomes

There was no significant difference in the resolution of fever (FCT) between the two treatment groups (Table 2).

**Table 2:** 

Outcome Type	Outcomes Sub-Categories	Treatment group (n = 287)		
		Gatifloxacin n = 145	Azithromycin n = 142	*p-*Value
Primary	Fever Clearance Time in hours (95% CI)	106 (94–118)	106 (88–112)	0.984 ˆ
Secondary	Overall treatment failure, numbers of patients (%)	13/145 (9)	13/140 (9.3)¶	0.854 ˆ
	Did not complete full treatment course, n (%)	0	2	
	*Clinical failure, n (%)	6/145 (4.3)	6/140 (4.2)	1.000#
	*Microbiological failure, n (%)	2/145 (1.4)	3/140 (2.2)	0.680#
	*Typhoid-fever related complications, n (%)	0/145 (0)	8/140 (5.7)	0.003#
	Gastrointestinal bleeding	0	4	
	Pneumonia	0	2	
	Liver dysfunction	0	2	
	Relapse after discharge from hospital, n (%)	4/137 (2.9)	0/127 (0)	0.052 ˆ
	§Number of patients with faecal carriage at follow-up, n (%)	1/137 (0.7)	0/131 (0)	

*Patients can fail in more than one subcategory.

¶ In the worst case scenario: 15/142 (10.6%) showed overall treatment failure in the azithromycin group, log rank test p = 0.570.

ˆThe *p* value is based on the log rank test.

# The *p* value is based on Fisher's exact test.

§ Evaluated in patients who attended at least two follow-up visits.

**Figure 2:**
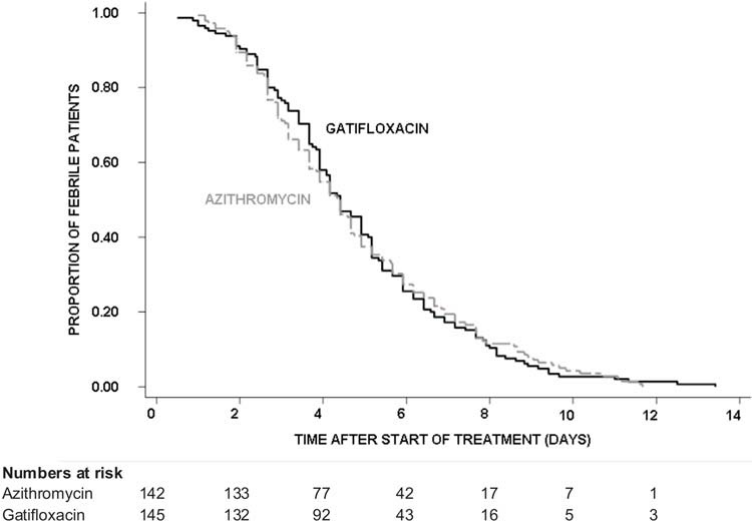
Proportion of culture confirmed patients still febrile. Kaplan-Meier survival curve showing the proportion of culture confirmed patients (PP analysis) still febrile through time by treatment group.

By PP analysis, the median FCT was 106 hours in both treatment arms (95% Confidence Interval [CI]; 94–118 hours for gatifloxacin *versus* 88–112 hours for azithromycin), (logrank test *p* = 0.984, HR [95% CI] = 1.0 [0.80–1.26]). The Kaplan-Meier survival curve for the fever clearance time is shown in Figure 2. At day 7, fever clearance rate was 82.8% (95% CI; 76.2%–88.4%) in the gatifloxacin group and 80.5% (95% CI; 73.6 %–86.6 %) in the azithromycin group.

**Figure 3:**
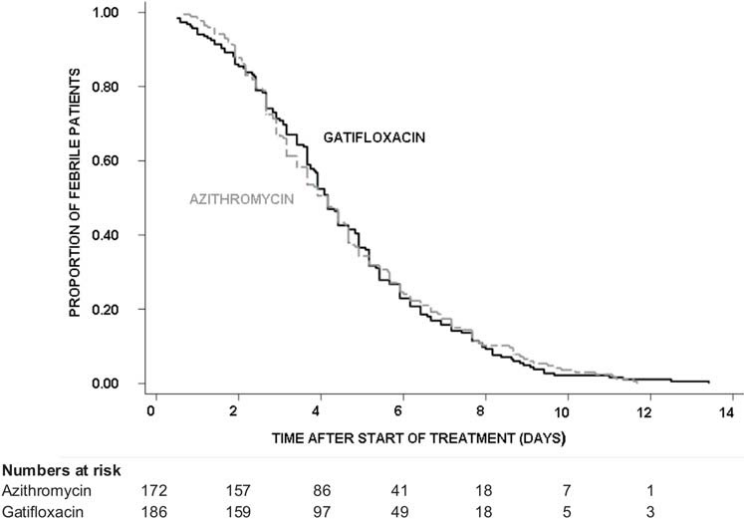
Proportion of all randomised patients still febrile. Kaplan-Meier survival curve showing the proportion of all randomised patients (ITT analysis) still febrile through time by treatment group.

In the ITT population, the median FCT was 100 hours in both treatment arms (95% CI; 92–106 hours for gatifloxacin *versus* 88–112 hours for azithromycin), (logrank test *p* = 0.914, HR [95% CI] = 1.01 [0.82–1.25]). At day 7, fever clearance rate was 84.2% (95% CI; 78.5%–89%) in the gatifloxacin group and 82.6% (95% CI; 76.5%–87.9%) in the azithromycin group (Figure 3).

#### Secondary outcomes

There was no death in the study.

There was no significant difference in overall failure to treatment between the two groups (Table 2).

**Figure 4:**
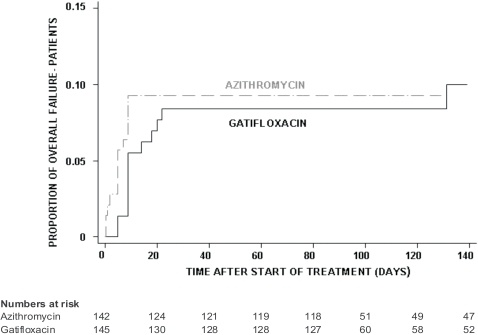
Proportion of patients with overall failure in the culture confirmed population. Kaplan-Meier survival curve showing the proportion of patients with overall failure in the culture confirmed population (PP analysis) by treatment group.

By PP analysis, the number of patients that showed overall failure to treatment was 13/145 (9%) in the gatifloxacin group and 13/140 (9.3%) in the azithromycin group (logrank test *p* = 0.854, HR [95% CI] = 0.93 [0.43–2.0]), or when assuming the worst case scenario, that all dropped-out patients were failures, 15/142 (10.6%) failures in the azithromycin group (logrank test *p* = 0.570, HR [95% CI] = 0.81 [0.38–1.7]). Figure 4 shows the proportion of patients failing through time after the start of treatment.

In the azithromycin arm, more than one failure event occurred in individual patients (Table 2). Clinical failure occurred in 6/145 (4.3%) patients in the gatifloxacin group and in 6/140 (4.2%) in the azithromycin group (*p* = 1.000, OR [95% CI] = 0.96 [0.25–3.7]). Three patients in each study arm were re-treated with ceftriaxone, the other patients resolved their symptoms within 24 hours.

Microbiological failure was seen in 2 out of 145 patients in the gatifloxacin arm (1.4%) and in 3 out of 140 (2.2%) in the azithromycin arm (*p* = 0.680, OR [95% CI] = 0.64 [0.05–5.7]). Two of the azithromycin recipients showed additionally signs of clinical failure.

There were no typhoid fever-related complications in the 145 gatifloxacin patients compared to 8 out of 140 (5.7%) patients in the azithromycin arm (*p* = 0.003, OR [95% CI] = 0 [0–0.4]). Two azithromycin recipients developed signs of liver dysfunction (elevated AST and ALT, deepening of jaundice) in addition to signs of clinical failure. Study treatment was continued and symptoms resolved by the time of discharge. Four patients, three children and one adult, suffered from gastrointestinal bleeding on day 3, day 5 (2 cases) and day 7 of treatment respectively, three patients received blood transfusions. One of these patients developed shock but responded to intravenous fluids and supportive treatment. Treatment was discontinued immediately in all the patients and re-treatment with ceftriaxone was initiated. Two adult patients developed pneumonia during treatment.

**Figure 5:**
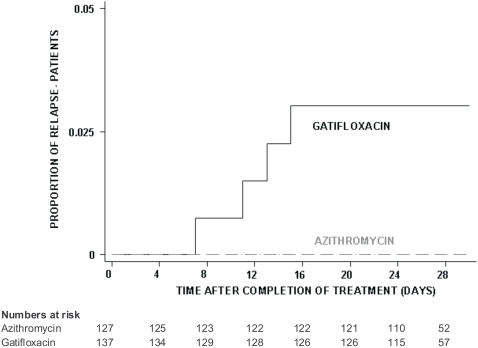
Proportion of patients with relapse in the culture confirmed population. Kaplan-Meier survival curve showing the proportion of patients with relapse in the culture confirmed population (PP analysis) by treatment group.

Relapse was evaluated only in patients that were initially categorised as successfully treated, patients with clinical failure, microbiological failure or complications were not evaluated. Four patients out of 137 (2.9%) relapsed in the gatifloxacin group compared to 0/127 in the azithromycin group (logrank test *p* = 0.052, HR [95% CI] = not estimable due to zero observations in one group), (Figure 5). These relapses with symptoms suggestive of typhoid fever occurred on day 7, 11, 13 and 15 respectively, after completion of treatment, three patients were confirmed culture positive for *S. typhi*. One patient developed acute respiratory distress syndrome (ARDS) and needed ventilation. The patient was treated with ceftriaxone and perfloxacine and subsequently made a complete recovery.

Chronic faecal carriage was evaluated in patients who attended at least two follow-up appointments, 137 in the gatifloxacin group and 131 in the azithromycin group. Only one patient with chronic faecal carriage was detected after 6 months (An Giang study site), the patient had received gatifloxacin.

In the ITT analysis (all 358 randomised patients), overall treatment failure was reported in 13 out of 185 (7%) in the gatifloxacin group compared to 14 out of 168 (8.4%) in the azithromycin group (logrank test *p* = 0.615, HR [95% CI] = 0.82 [0.39–1.76]). One culture negative patient in the azithromycin group had a positive blood culture on day 7 after start of treatment. There were no clinical failures or typhoid fever-related complications in the culture negative patients.

### Adverse events

Both treatments were well tolerated. One adverse event related to azithromycin was reported, a maculopapular rash that occurred after the first dose of treatment. Azithromycin was discontinued immediately and the patient was treated with ceftriaxone.

Gastrointestinal side-effects (change in consistency and frequency of stools) that were probably typhoid fever related were relatively frequent in both treatment arms at the start of treatment. In the gatifloxacin group, one patient experienced vomiting on day 2 and day 3 and one patient diarrhoea (4 episodes/day) on day 4 and day 5 of treatment. These episodes were self-limiting and did not require the interruption of therapy.

The median levels of serum AST and ALT fell in both groups after 7 days of therapy. In the PP group, the median post-treatment AST was 46.35 U/L (range 12.8–217.5) in the gatifloxacin arm and 45 U/L (range 5–358) in the azithromycin arm. The median post-treatment ALT fell to 46.8 U/L (range 7.4–278) and 49.9 (1.1–494), respectively. In the culture-negative patients, the median post-treatment AST was 44.8 U/L (range 12–654) and ALT was 40 U/L (range 10–424.4).

### Antimicrobial susceptibilities of *S. typhi* and *S. paratyphi* A isolates

From the PP population, 282 (98%) *S. typhi* and 5 (2%) *S. paratyphi* A strains were isolated. Two hundred and sixty three *S. typhi* and 5 *S. paratyphi* A were received at the Hospital for Tropical Diseases for antimicrobial susceptibility testing.

Fifty-eight percent of the *S. typhi* isolates were MDR and 96% were nalidixic acid resistant and showed reduced susceptibility to the older generation fluoroquinolones (Table 3). However technically, using current CLSI breakpoints, all isolates remained susceptible in vitro to ciprofloxacin and ofloxacin. The MIC_90_ of gatifloxacin was the lowest of all the fluoroquinolones tested at 0.19 µg/mL (range 0.004–0.5). All isolates were susceptible to ceftriaxone.

**Table 3:** Antimicrobial susceptibilities and minimum inhibitory concentrations (MIC) of 263 *S. typhi* isolate.

			Treatment with	
		All isolates	Gatifloxacin	Azithromycin
		n = 263	n = 137	n = 126
Multidrug resistant, numbers (%)		153 (58)	87 (63.5)	66 (52.3)
Nalidixic acid resistant, numbers (%)		254 (96.5)	132 (96.3)	121 (96)
	MIC 50 (µg/ml)	>256	>256	>256
Amoxicillin	MIC 90 (µg/ml)	>256	>256	>256
	range (µg/ml)	0.125 to >256	0.5 to >256	0.125 to >256
	MIC 50 (µg/ml)	>256	>256	>256
Chloramphenicol	MIC 90 (µg/ml)	>256	>256	>256
	range (µg/ml)	0.38 to >256	2 to >256	0.38 to >256
	MIC 50 (µg/ml)	>256	>256	>256
Nalidixic acid	MIC 90 (µg/ml)	>256	>256	>256
	range (µg/ml)	1.5 to >256	1.5 to >256	1.5 to >256
	MIC 50 (µg/ml)	0.75	0.75	1
Ofloxacin	MIC 90 (µg/ml)	1.5	1.5	1.5
	range (µg/ml)	0.023–2	0.032–2	0.023–2
	MIC 50 (µg/ml)	0.38	0.38	0.38
Ciprofloxacin	MIC 90 (µg/ml)	0.5	0.5	0.5
	range (µg/ml)	0.004–0.75	0.006–0.75	0.004–0.38
	MIC 50 (µg/ml)	0.125	0.125	0.125
Gatifloxacin	MIC 90 (µg/ml)	0.19	0.19	0.19
	range (µg/ml)	0.004–0.5	0.006–0.25	0.004–0.5
	MIC 50 (µg/ml)	0.125	0.125	0.125
Ceftriaxone	MIC 90 (µg/ml)	0.125	0.125	0.19
	range (µg/ml)	0.064–0.25	0.064–0.19	0.064–0.25
	MIC 50 (µg/ml)	8	8	8
Azithromycin	MIC 90 (µg/ml)	12	12	12
	range (µg/ml)	1.5–16	1.5–16	4–16

MIC_50/90_, concentration at which 50% and 90% of the organisms respectively are inhibited. MDR is defined as resistance to chloramphenicol, ampicillin and trimethoprim-sulfamethoxazole. CLSI MIC breakpoints are as follows: for chloramphenicol, ampicillin and nalidixic acid resistance ≥32 µg/mL; ofloxacin and gatifloxacin ≤2 µg/mL susceptible and ≥8 µg/mL resistant; ciprofloxacin ≤1 µg/mL susceptible and ≥4 µg/mL resistant; ceftriaxone ≤8 µg/mL susceptible and ≥64 µg/mL resistant; there are none for azithromycin.

The 5 *S. paratyphi* A strains were fully susceptible to all the antimicrobials tested.

## Discussion

### Interpretation

The results of this trial show that both antibiotics worked well for the treatment of MDR and nalidixic acid resistant typhoid fever in Vietnam. A seven day oral course of gatifloxacin had similar efficacy and safety as a seven day course of azithromycin, which is recommended for the treatment of MDR and nalidixic acid resistant typhoid fever [7], [9].

However, azithromycin is not available throughout much of the developing world and it is expensive. The costs of a 7-day treatment course of gatifloxacin (at 10 mg/kg/day) for an adult patient in Vietnam are approximately 25 US$, the costs of azithromycin (at 20 mg/kg/day) are more than 90 US$.

The results for gatifloxacin in this trial are comparable to the excellent clinical outcomes achieved with ofloxacin in Vietnam in the early 1990s, when *S. typhi* isolates were still susceptible to nalidixic acid [17]–[19].

Gatifloxacin has a higher affinity to GyrA and is less inhibited by the common mutations in the *GyrA* gene [20]. The gatifloxacin MIC_50_ of the study isolates was 0.19 µg/mL compared to the oxfloxacin MIC_50_ of 0.75 µg/mL. We would not recommend the continued use of the older generation fluoroquinolones (ofloxacin and ciprofloxacin) in regions with high rates of nalidixic acid resistant typhoid fever for fear of selecting further mutations in *gyrA* [21]. This could put at risk the potential clinical benefit of the newer fluoroquinolones, including gatifloxacin.

There have been several case reports of gatifloxacin-associated dysglycemia in patients with type II diabetes mellitus, overweight or with other comorbidity [22]–[24]. Recently there have been concerns about the use of gatifloxacin, after a retrospective case-control study in 1.4 million individuals over the age of 66 years (mean age 77 years) in Canada was published [25].

As our trial was completed before publication of this report, we did not systematically monitor for hypo- and hyperglycemia. Blood glucose levels taken as part of the routine care were normal. All patients were managed as in-patients and potential symptoms of hypo- and hyperglycemia would have been noted by the study physicians. No dysglycemia events were reported during the in-patient period or during the follow up period of 3 to 6 months.

The patients in our trial were healthy, young and non-obese individuals. A trial in 867 children with otitis media with glucose monitoring and a one year follow-up [26], as well as a recent enteric (typhoid and paratyphoid) fever trial in Nepal used gatifloxacin and did not report any dysglycemia [10]. In our setting and in our patient population gatifloxacin was highly effective despite very high rates of drug resistance and was well tolerated.

Other newer generation fluoroquinolones, i.e. gemifloxacin and moxifloxacin have shown low MICs for nalidixic acid resistant *S. typhi* and *S. paratyphi A* [11], unfortunately these drugs are not available in Vietnam and they are considerably more expensive. The in vitro results seen with these other newer generation fluoroquinolones should be evaluated in clinical trials.

### Generalizability

The emergence of nalidixic acid resistant *S. typhi* and *S. paratyphi* A with reduced susceptibility to the fluoroquinolones is a widespread problem throughout Asia and therefore our study is relevant to the whole region [2], [6]. Many case reports and some randomised controlled trials have described the worsening clinical response to ciprofloxacin and ofloxacin [8], [27], [28].

The search for effective antibiotics to treat typhoid fever is imperative.

Typically trials in typhoid fever are limited by small sample sizes, a recent Cochrane Report has stressed the need for large well-designed trials in enteric fever [29]. The evidence from our trial is strengthened by a sample size of 287 patients with culture confirmed typhoid fever (358 patients randomised), which we believe is so far the largest RCT performed in typhoid fever.

Both antibiotics also worked well for the patients with negative blood cultures. This is an important finding because the sensitivity of blood culture for the diagnosis of typhoid fever is only approximately 50 to 80% [9].

### Limitations of the study

The randomisation sequence was generated with a large block size of 50, which resulted in uneven numbers in the two treatment groups (186 versus 172 patients in the ITT population).

One possible limitation was the low rate of stool cultures positive for *S. typhi.* Faecal carriage is usually characterised by intermittent shedding and the stool culture for *S. typhi* is not very sensitive. When comparing our data with other studies that demonstrate that azithromycin is highly efficacious for the treatment of typhoid fever, we find similar low rates of faecal carriage at follow-up [7], [30]. It could be hypothesized that antibiotics that show high intracellular concentrations and good tissue penetration like azithromycin and the fluoroquinolones, achieve rapid bacterial killing and elimination throughout the body, which reduces faecal carriage.

The dose of gatifloxacin and azithromycin tablets was prepared by careful cutting of the tablets (proportions of the tablets administered were recorded in the CRFs). Inevitably, it was therefore an estimation of the exact dose, hence we cannot guarantee that each patient received exactly 10 mg/kg/day of gatifloxacin or 20 mg/kg/day of azithromycin.

#### Overall evidence

We performed a MEDLINE search for “azithromycin, clinical trial, typhoid/enteric fever” and used the recent enteric fever Cochrane report [29] to identify 6 clinical trials in the literature. In total, 251 typhoid fever patients were treated with azithromycin.

Four trials, three from Egypt and one from India, used azithromycin to treat MDR typhoid fever [30]–[33]. Azithromycin achieved cure rates between 88% and 100%, the mean FCT ranged from 3.8 to 4.5 days. Two trials performed in Vietnam used azithromycin at 20 mg/kg/day [7] and at 10 mg/kg/day [8] for the treatment of MDR and nalidixic acid resistant typhoid fever. In total, 107 patients with culture confirmed typhoid fever were enrolled. The cure rate was 93% and 82% and the FCT was 5.6 and 5.8 days, respectively. Our results concur with these excellent data .

A recent trial conducted in Kathmandu, Nepal used gatifloxacin at the same dose and duration for the treatment of nalidixic acid resistant typhoid fever [10]. Successful treatment was achieved in 96.5% (85 out of 88) patients and the median FCT (95% CI) was 92 hours (84–114 hours). The trial in Nepal was stopped early by the independent Data and Safety Monitoring Committee as a result of the poor clinical response in the patients randomised to cefixime.

We believe on the basis of this and other recently published trials, that gatifloxacin or azithromycin are now the treatments of choice for enteric fever in areas of MDR and nalidixic acid resistance [7], [8], [10]. However it is important to use these antimicrobial agents cautiously because indiscriminate use would inevitably induce further resistance.

## Supporting Information

Protocol S1 Trial Protocol
(0.07 MB DOC)

Checklist S1 CONSORT Checklist
(0.06 MB DOC)
