# Supplementary material for: A Multi-Center Randomised Controlled Trial of Gatifloxacin versus Azithromycin for the Treatment of Uncomplicated Typhoid Fever in Children and Adults in Vietnam
Source: PLoS One. 2008 May 21;3(5):e2188. doi: 10.1371/journal.pone.0002188 (PMC2374894; doi:10.1371/journal.pone.0002188)
Supplement: Protocol S1 — Trial Protocol (0.07 MB DOC) [file pone.0002188.s001.doc]

**An open randomized comparison of gatifloxacin versus azithromycin for the treatment of uncomplicated enteric fever**

**Study Code: BJ**

**1. Institution and Department**

Dong Thap Hospital

An Giang Hospital

Hospital for Tropical Diseases Ho Chi Minh City

University of Oxford Clinical Research Unit Ho Chi Minh City Viet Nam

**3. Project Title**

An open randomized comparison of gatifloxacin versus azithromycin for the treatment of uncomplicated enteric fever

**Backgound**

Recent treatment results from the Hospital for Tropical Diseases and Dong Thap Provincial Hospital in uncomplicated nalidixic acid resistant enteric fever suggests a 30% failure rate with the fluoroquinolones (Ofloxacin or Ciprofloxacin) at a dose of 20 mg/kg/day for 10-14 days and a fever clearance time of >300 hours. Short and longer term convalescent faecal excretion of *S.* Typhi can also be high with this regimen and this potentially leads to high levels of secondary transmission in the community following apparent cure.

Gatifloxacin is a broad spectrum synthetic 8-methoxyfluoroquinolone. After a single 400mg the Cmax is 4.2μg/ml with an AUC of 51.3. It is known to be concentrated in macrophages at a intracellular;serum ratio of 26.5 (10.9-61.1). Comparative MICs for Styphi are given below:

| strain | Ofloxacin | Levofloxacin | gatifloxacin |
| --- | --- | --- | --- |
| 1 | 0.75 | 0.25 | 0.19 |
| 2 | 0.75 | 0.25 | 0.19 |
| 3 | 0.047 | 0.016 | 0.012 |
| 4 | 0.5 | 0.25 | 0.19 |
| 5 | 0.75 | 0.25 | 0.19 |
| 6 | 1.5 | 0.5 | 0.25 |
| 7 | 0.75 | 0.25 | 0.19 |
| 8 | 2 | 0.75 | 0.38 |
| ATCC Ecoli | 0.065 | 0.016 | 0.012 |

Of the available fluoroquinolones Gatifloxacin has the lowest MIC. Unpublished data has suggested a mean FCT of 76 hours when used in a small number of patients with S typhi (8 NaliR isolates and 2 NaliS isolate). Gatifloxacin is available, affordable and registered in Viet Nam.

Azithromycin has given 95 percent cure rates following 5 to 7 day treatment courses. The mean FCT is 166 hours and relapse and convalescent faecal carriage rates are less than 3 percent. However it is not widely available, in vitro resistance has been shown to be developing and it is expensive. It is however (arguably) currently the best therapy for MDR and NaliR typhoid fever in Viet Nam.

There is an urgent need for a treatment that combines speed of clinical response, and reduction in secondary transmission. Quinolone resistance is usually mediated by single point mutations in the quinolone resistance determining region of the *gyr*A gene characteristically occurring at position 83 of the DNA gyrase enzyme (changing serine to phenyalanine) and position 87 (changing aspartate to tyrosine or glycine). In other Enterobacteriaciae, higher levels of quinolone resistance have been associated with additional mutations in the *gyr*A gene, in other topoisomerase genes, or alterations in fluoroquinolone uptake. No such mutations have been reported yet in *S*.Typhi, although if they were to occur it is likely that it would result in full fluoroquinolone resistant isolates. Gatifloxacin is a broad spectrum synthetic 8-methoxyfluoroquinolone whose mode of action is slightly different to the other fluoroquinolones and as such is less susceptible to the common mutations found in the Salmonella typhi isolates in Viet Nam. This may explain the lower MICs and anecdotally faster clinical responses. This anecdotal reports need to be verified before Gatifloxacin can be recommended for widespread use.

Both Azithromycin and the fluoroquinolones have been shown to be effective in typhoid fever. Azithromycin is the current treatment of choice although there is little experience in children and there has been in vitro evidence of resistance developing amongst Enterobacteriaciae. Without new treatment options becoming available for typhoid fever in Viet Nam it is likely that in the near future we will see the emergence of untreatable typhoid fever and a return to the pre-antibiotic era. This open randomised study aims to compare the clinical response to a combination of Gatifloxacin versus Azithromycin for the treatment of uncomplicated typhoid fever

#### Aim

A single open randomized comparison of Gatifloxacin and Azithromycin for 7 days in patients with uncomplicated typhoid fever.

**Patient selection:**

Patients will be eligible to be admitted to the study if they have suspected or culture proven enteric fever provided:

1. They give fully informed consent.
2. They are not obtunded or shocked, are not deeply jaundiced and have no signs of gastrointestinal bleeding or any other evidence of severity.
3. No previous history of hypersensitivity to either of the trial drugs
4. No known previous treatment with a quinolone antibiotic or 3rd generation cephalosporin or macrolide within one week of hospital admission. (Patients who have received chloramphenicol, ampicillin, or co-trimoxazole will be included as long as they have not shown evidence of clinical response).
5. They are not pregnant.
6. Age over 6 months.

#### Treatment

This will be an open, randomised comparison. The treatment allocation will be in an opaque envelope. Once the envelope is opened the patient will be considered to have entered the trial whatever happens subsequently. Patients will be randomized to group A or group B.

Group A: Gatifloxacin 10mg/kg/day for 7 days

GroupB: Azithromycin 20mg/kg/day for 7 days

#### Observations and Investigations

a) Prior to admission to the study

1. Full history and clinical examination. In particular the following data will be documented: Clinical manifestations

- Fever clearance time
- Any side effects of the drug
- Complications of the disease (if any occur)

1. Chest X-ray and other radiological investigations, including abdominal ultrasound, as clinically indicated.
2. Hct, white cell count/differential, platelet count, SGOT, SGPT, CRP and urinalysis
3. Microbiology:

- Blood cultures (5-8 ml)
- Urine dipstick
- Stool culture x 3 pre-treatment
- Bone marrow will be performed where possible on patients with suspected enteric fever whose blood culture is negative after 48 hours.

# **Day 1 - Admission to study**

- Axillary temperature will be recorded at 6 hourly intervals and a daily symptom and activity record will be kept.

## **Day 7 (At the end of treatment)**

- Hct, white cell count, platelets, SGOT, SGPT will be repeated on day 9 (before discharge) or as indicated by results and clinical condition.
- Blood cultures will be performed on day 9. Faeces will be re-cultured on day 8, 9 or 10.
- Abnormal radiological investigations will be repeated as appropriate.
- All isolates from the initial illness, “relapse” and asymptomatic carriage on follow-up will be stored at - 20^0^C for antibiotic susceptibility testing, plasmid typing and pulse field gel electrophoresis to distinguish relapse and re-infection. ]

**Out-patient follow up –One months**

1. Full history and relevant examination will be performed. Any previously abnormal laboratory investigation will be repeated, stool culture
2. A full set of microbiological cultures (blood) will only be performed if symptoms and signs suggest further infection.
3. If patients do not attend follow up a member of the study team will visit their home to find out about clinical symptoms and obtain at least one stool specimen.
4. Patients with convalescent stool carriage of S.typhi or S.paratyphi A will have arrangement will be made for further follow up. An ultrasound will be performed to exclude biliary or kidney stones if carriage is persistent.
5. If carriage is present 4 to 6 weeks following treatment a further course of treatment will be given according to the antibiotic sensitivity of the isolate.

**Out-patient follow up** - Three month

- Full history and relevant examination will be performed.
- Any previously abnormal laboratory investigation will be repeated. Stool culture x 1.
- A full set of microbiological cultures (blood) will only be performed if symptoms and signs suggest further infection.
- If patients do not attend follow up they will be reminded by letter or a member of the team will visit their home to ask about clinical symptoms and obtain at least one stool specimen.

**Out-patient follow up** –Six months

- Full history and relevant examination will be performed.
- Any previously abnormal laboratory investigation will be repeated. Stool culture x 1.
- A full set of microbiological cultures (blood) will only be performed if symptoms and signs suggest further infection.
- If patients do not attend follow up they will be reminded by letter or a member of the study team will visit their home to ask about clinical symptoms and obtain at least one stool specimen.

#### Treatment outcome assessment

The response to treatment will be assessed by

Primary:

FEVER CLEARANCE TIME

(Time to first fall ≤ 37.5^0^C, axillary, and to remain ≤ 37.5^0^C for 48 hours)

Secondary:

TREATMENT FAILURES

- Clinical Failure: persistence of fever and symptoms for > 2 days after the end of treatment (ie on day 10)
- The development of any severe complication.
- Microbiological failure: positive blood culture on day 9.
- Need for retreatment
- Occurrence of relapse of infection at follow up
- Occurrence of faecal carriage at follow-up visits (to exclude faecal carriage a minimum of two consecutive follow-up visits had to be attended).

#### Failure of treatment or relapse

Patients who fail will be retreated with ceftriaxone 2g/day for 10 days.

#### Removal from the study

Patients who fail treatment or have organisms other than S.typhi or S.paratyphi A grown from admission blood will be removed from the study and given appropriate treatment, but their outcome will be recorded.

#### Adverse events

All adverse events will be fully recorded including duration, severity, outcome and relationship to study drug. Liver function tests will be repeated on day 8 in all patients. If abnormal further tests will be performed on day 10 or until they return to normal.

#### Samples stored

1. All Isolates
2. Plasma/EDTA sample on admission
3. Plasma/EDTA sample on discharge

# **Support**

The OUCRU-VN will provide the drugs and support for the trial.

**PATIENT CHECKLIST**

1. **PATIENT THOUGHT TO HAVE TYPHOID FEVER □**

##### CONSENT TO TRIAL □

##### RANDOMISED □

1. **BLOOD CULTURE □**

**BLOOD INVESTIGATIONS □**

1. **EVERY SIX HOURS TEMPERATURE AND ASSESSMENT □**
2. **AFTER 9 DAYS REPEAT BLOOD CULTURE □**

**BLOOD INVESTIGATIONS □**

1. **STOOL CULTURE DAYS 8, 9, OR 10 □**
2. **FOLLOW UP ONE MONTH □**
3. **FOLLOW UP THREE MONTHS**
4. **FOLLOW UP SIX MONTHS □**
