# Supplementary material for: A Multi-Center Randomised Controlled Trial of Gatifloxacin versus Azithromycin for the Treatment of Uncomplicated Typhoid Fever in Children and Adults in Vietnam
Source: PLoS One. 2008 May 21;3(5):e2188. doi: 10.1371/journal.pone.0002188 (PMC2374894; doi:10.1371/journal.pone.0002188)
Supplement: Checklist S1 — CONSORT Checklist (0.06 MB DOC) [file pone.0002188.s002.doc]

| *PAPER SECTION* And topic | Item | Description | Reported in section # |
| --- | --- | --- | --- |
| *TITLE & ABSTRACT* | 1 | [How participants were allocated to interventions](http://www.consort-statement.org/Explanation/examples1.htm) (*e.g*., "random allocation", "randomized", or "randomly assigned"). | Abstract |
| *INTRODUCTION* Background | 2 | [Scientific background and explanation of rationale.](http://www.consort-statement.org/Explanation/examples2.htm) | Introduction |
| *METHODS* Participants | 3 | [Eligibility criteria for participants](http://www.consort-statement.org/Explanation/examples3a.htm) and the [settings and locations where the data were collected](http://www.consort-statement.org/Explanation/examples3b.htm). | Participants,  Study sites |
| Interventions | 4 | [Precise details of the interventions intended for each group and how and when they were actually administered.](http://www.consort-statement.org/Explanation/examples4.htm) | Interventions,  Procedures |
| Objectives | 5 | [Specific objectives and hypotheses](http://www.consort-statement.org/Explanation/examples5.htm). | Study design and objectives |
| Outcomes | 6 | [Clearly defined primary and secondary outcome measures](http://www.consort-statement.org/Explanation/examples6a.htm) and, when applicable, any [methods used to enhance the quality of measurements](http://www.consort-statement.org/Explanation/examples6b.htm) (*e.g.*, multiple observations, training of assessors). | Outcomes of the study |
| Sample size | 7 | [How sample size was determined](http://www.consort-statement.org/Explanation/examples7a.htm) and, when applicable, [explanation of any interim analyses and stopping rules](http://www.consort-statement.org/Explanation/examples7b.htm). | Sample size,  Study site and ethical approval |
| Randomization -- Sequence generation | 8 | [Method used to generate the random allocation sequence](http://www.consort-statement.org/Explanation/examples8a.htm), including [details of any restriction](http://www.consort-statement.org/Explanation/examples8b.htm) (*e.g.*, blocking, stratification). | Randomisation procedures |
| Randomization -- Allocation concealment | 9 | [Method used to implement the random allocation sequence](http://www.consort-statement.org/Explanation/examples9.htm) (*e.g*., numbered containers or central telephone), clarifying whether the sequence was concealed until interventions were assigned. | Randomisation procedures |
| Randomization -- Implementation | 10 | [Who generated the allocation sequence, who enrolled participants, and who assigned participants to their groups.](http://www.consort-statement.org/Explanation/examples10.htm) | Randomisation procedures |
| Blinding (masking) | 11 | [Whether or not participants, those administering the interventions, and those assessing the outcomes were blinded to group assignment.](http://www.consort-statement.org/Explanation/examples11a.htm) When relevant, [how the success of blinding was evaluated](http://www.consort-statement.org/Explanation/examples11b.htm). | Study design, Blinding |
| Statistical methods | 12 | [Statistical methods used to compare groups for primary outcome(s)](http://www.consort-statement.org/Explanation/examples12a.htm); [Methods for additional analyses,](http://www.consort-statement.org/Explanation/examples12b.htm) such as subgroup analyses and adjusted analyses. | Statistical methods |
| RESULTS  Participant flow | 13 | [Flow of participants through each stage](http://www.consort-statement.org/Explanation/examples13a.htm) (a diagram is strongly recommended). Specifically, for each group report the numbers of participants randomly assigned, receiving intended treatment, completing the study protocol, and analyzed for the primary outcome. [Describe protocol deviations from study as planned, together with reasons.](http://www.consort-statement.org/Explanation/examples13b.htm) | Flow of participants and recruitment,  Figure 1 |
| Recruitment | 14 | [Dates defining the periods of recruitment and follow-up.](http://www.consort-statement.org/Explanation/examples14.htm) | Flow of participants and recruitment |
| Baseline data | 15 | [Baseline demographic and clinical characteristics of each group.](http://www.consort-statement.org/Explanation/examples15.htm) | Baseline data,  Table 1 |
| Numbers analyzed | 16 | [Number of participants (denominator) in each group included in each analysis and whether the analysis was by "intention-to-treat"](http://www.consort-statement.org/Explanation/examples16.htm) . State the results in absolute numbers when feasible (*e.g*., 10/20, not 50%). | Numbers analysed |
| Outcomes and estimation | 17 | [For each primary and secondary outcome, a summary of results for each group, and the estimated effect size and its precision](http://www.consort-statement.org/Explanation/examples17.htm) (*e.g.*, 95% confidence interval). | Outcomes and estimation, Table 2 |
| Ancillary analyses | 18 | [Address multiplicity by reporting any other analyses performed](http://www.consort-statement.org/Explanation/examples18.htm), including subgroup analyses and adjusted analyses, indicating those pre-specified and those exploratory. | Non applicable |
| Adverse events | 19 | [All important adverse events or side effects in each intervention group.](http://www.consort-statement.org/Explanation/examples19.htm) | Adverse events |
| DISCUSSION Interpretation | 20 | [Interpretation of the results](http://www.consort-statement.org/Explanation/examples20.htm), taking into account study hypotheses, sources of potential bias or imprecision and the dangers associated with multiplicity of analyses and outcomes. | Interpretation |
| Generalizability | 21 | [Generalizability (external validity) of the trial findings.](http://www.consort-statement.org/Explanation/examples21.htm) | Generalizability |
| Overall evidence | 22 | [General interpretation of the results in the context of current evidence.](http://www.consort-statement.org/Explanation/examples22.htm) | Overall evidence |
